# Comparative Medicine in the Twenty-First Century: Where are We Now and Where Do We Go from Here?

**DOI:** 10.3389/fvets.2015.00002

**Published:** 2015-05-21

**Authors:** Ali Mobasheri

**Affiliations:** ^1^School of Veterinary Medicine, Faculty of Health and Medical Sciences, University of Surrey, Guildford, UK; ^2^Center of Excellence in Genomic Medicine Research (CEGMR), King AbdulAziz University, Jeddah, Saudi Arabia; ^3^King Fahd Medical Research Center (KFMRC), King AbdulAziz University, Jeddah, Saudi Arabia

**Keywords:** grand challenge, comparative medicine, one health, one medicine, translational medicine, interdisciplinary collaboration, communication

“Comparative Medicine” may be defined as a field of study concentrating on similarities and differences between veterinary medicine and human medicine. However, this is a redundant definition that needs to be updated, modernized, and refined. The nineteenth century German physician Rudolf Ludwig Carl Virchow, widely known as the “Father of Modern Pathology” and the founder of the science of veterinary pathology wrote:

Between animal and human and medicine, there is no dividing line – nor should there be. The object is different, but the experience obtained constitutes the basis of all medicine.

In his 1927 paper entitled “What is Comparative Medicine?” published in the Proceedings of the Royal Society of Medicine (1), O. Charnock Bradley defined “Comparative Medicine” as two branches of “One Medicine.” He wrote:

Inasmuch as it includes the study of disease in a considerable number of animals belonging to widely different species, there is some ground for regarding veterinary medicine as being comparative medicine. But this is held to be too narrow an application of the term.

Clearly, even by the late 1920s the definition of “Comparative Medicine” was considered too narrow and inadequate. Bradley went further by proposing:

Human and veterinary medicine are confronted with similar problems and employ similar means for their solution; and, taken together, they deal with a large group of animals sufficient to justify the contention that they are two branches of one medicine.

## The Evolving Concept of One Medicine

The concept of “One Medicine” emerged in 1970s and is credited to Dr. Calvin Schwabe (1927–2006), a veterinary epidemiologist and parasitologist in his textbook “Veterinary Medicine and Human Health” (2). “One Medicine” eventually gave rise to the present “One Health” initiative, which aims to unite human and veterinary medicine. However, it is important to point out that in the decades and centuries prior to the emergence of the “One Health, One Medicine” concept, “Comparative Medicine” was already recognized as a field of study within medicine, a field that was synonymous with “laboratory animal medicine compared with human medicine.” The twentieth century witnessed enormous advances in “Comparative Medicine” and numerous biomedical research contributions involved the use of laboratory animals (i.e., rodents, non-human primates, dogs, cats, and more recently, new laboratory species such as the zebrafish). Discussion of these contributions is beyond the scope of this Specialty Grand Challenge article and will be the subject of a future paper in this section of Frontiers in Veterinary Science.

## Mice are Not Men

It is pertinent to mention the many limitations and challenges of relying on laboratory animals as models of human disease. Clearly, “mice are not men” and information obtained from mouse models does not always translate well to humans. Rodents are not always reliable as preclinical models for human disease. Mice have traditionally been used in immunology but recent studies have shown that mice do not reproduce the patterns of gene expression induced by human inflammatory disease (3). The scientific literature and clinical trial databases are littered with numerous examples of drugs that worked well in laboratory animals but turned out to be ineffective in clinical trials on humans. For example, in 1990s, cancer and arthritis were regarded as the most suitable diseases for the use of MMP inhibitors (MMPIs) and although results from animal studies suggested that MMP inhibition could be an effective therapeutic approach, clinical trials in humans failed and seriously diminished interest in MMP inhibition as a valid therapeutic option (4). MMPIs are not the only examples; there are many others in the literature. Such failures have been significant and extremely costly to the global pharmaceutical industry. These findings have provoked renewed discussion and debate concerning the validity of animal models in translational research (5). Researchers in favor of mouse models insist that gene expression patterns in mouse models closely align to those in human inflammatory conditions and continue to argue for the utility of mice as animal models of human disorders (6). Despite the drug development failures mice undoubtedly have an important place in basic research, preclinical testing, and the development of translational research pathways. In order to facilitate translational research, the use of chimeric rodents and humanized mouse models has been proposed (7, 8). Clearly, there are other obligations involved here since regulatory authorities require certain species to be used in toxicity as well as efficacy profiling. However, it is always important to recognize the inherent weakness and limitation of mouse models and we hope that contributors to Frontiers in Veterinary Science will submit papers relevant to this important topic.

## Justifying the Use of Animal Models

There is increasing pressure on researchers to defend and justify the validity of their animal models. Researchers, funding bodies, and publishers have been advised to pay more attention to the “3Rs” (replacement, refinement, and reduction of animals in research). Guidelines have been developed by the National Centre for the Replacement, Refinement, and Reduction of Animals in Research (NC3Rs) in the United Kingdom to improve the reporting of research using animals in order to maximize information published and minimize unnecessary studies. These guidelines, known as animal research: reporting of *in vivo* experiments (ARRIVE) were developed in consultation with the scientific community as part of the NC3Rs initiative to improve the standard of reporting of research using animals (9). These initiatives have led to the refinement of the definition of “Comparative Medicine” and how “One Health” may encompass it. Unfortunately, almost 5 years after the publication of the ARRIVE guidelines, very few journals and funding bodies are actually enforcing these guidelines and there has been very little improvement in reporting standards since then (10). Clearly, there is still a huge amount of work that needs to be done in order to encourage the entire scientific community to be more transparent about animal research and engage more openly with the general public. The European Commission has released guidance and issued legislation for the protection of animals used for scientific purposes (http://ec.europa.eu/environment/chemicals/lab_animals/legislation_en.htm).

## Shared Heritage of One Medicine

Despite a century of achievements and advances in “One Health, One Medicine” and “Comparative Medicine,” we have made scant progress and learned very little from Virchow, Bradley, and the scholars that followed in their footsteps. “One Health, One Medicine” has largely failed to realize its true potential in the twentieth century (11). The reason for this failure is quite simple and does not need to be explained using complex mathematical and statistical techniques: veterinary and medical sciences do not interact enough. The veterinary and medical professions are steeped in a rich history of “One Medicine,” but they have paradoxically parted ways (12). Veterinarians and medical practitioners still do not recognize that they speak different dialects of the same language. With the exception of the annual experimental biology meeting, which is organized by the Federation of American Societies for Experimental Biology (FASEB) veterinarians and physicians rarely attend the same conferences, conceive concepts for grant proposals and co-author scientific papers.

## Promoting Multidisciplinary Collaboration

The author concedes that there are numerous examples where veterinarians and physicians collaborate successfully and productively. However, these are exceptions rather than the rule. Indeed, there are many examples where veterinary, medical, dental, and bioscience schools within the same academic institution are separated by miles and located in separate campuses. Interestingly, there is evidence that the fruitful collaborations occur in institutions where veterinary, medical, and biomedical faculties co-exist in the same campuses and buildings (examples, Duke University, University of Pennsylvania, Western University of Health Sciences, Boston University, Tufts University, University of California, Berkeley and Davis). In these institutions, “Comparative Medicine” is a thriving multidisciplinary discipline where veterinarians, physicians, biomedical scientists, and researchers from physical sciences, engineering, and humanities focus on fundamental biomedical questions. By studying pathologies that co-exist in humans and animals, we are much more likely to uncover common denominators of disease and identify new therapeutic targets.

However, “Comparative Medicine” is not just about common mechanisms of disease and has the potential to transform and revolutionize translational medicine (the bench to bedside paradigm) and provide real-life solutions to unresolved challenges in healthcare. And as the discipline evolves new opportunities arise to reduce, refine, and replace animals in biomedical research and boost the drug discovery and development pipelines.

## Facilitating Drug Development

The global pharmaceutical industry, governments, and the major funding bodies recognize the need for interdisciplinary collaboration. New funding schemes are increasingly placing greater emphasis on interdisciplinarity due to the realization that societal challenges in health are unlikely to be met by medical researchers alone. If we are to tackle complex diseases such as cancer, we need to think outside the box. Veterinarians and physicians need to talk to each other as well engineers, mathematicians, and social scientists. They need to seek advice from physicists and statisticians. Multidisciplinary collaboration is the only way to ensure that accelerating advances in basic science can be translated into diagnostics, therapeutics, and most important of all, new preventive strategies for the most common and hard to treat diseases in the twenty-first century.

In terms of drug discovery and development, most of the low-hanging fruit is gone. The challenge is to reach higher and tackle polygenic diseases with complex etiologies using multidisciplinary approaches. To reap the rewards, we need to leave our preconceived prejudices behind, communicate and collaborate openly and more effectively. Today’s veterinary and medical graduates are truly outstanding, but they have the potential to be even better. To achieve this, they need not compete – they simply need to communicate and collaborate. They must communicate openly, share their knowledge, and discard their preconceptions and prejudices, especially when they deal with the public, politicians, and scientists without clinical qualifications. Many veterinary schools have begun initiatives to encourage their students to spend time with other stakeholders (e.g., funding agencies, research organizations, government departments, organizations, and pharmaceutical companies).

## Meeting the Challenges of Big Data

In order to meet the health challenges of the twenty-first century, we need to change the way research and development is carried out. We must first accept that in order to make informed decisions about health and health care choices, we need high-quality data – or “big data.” Collecting “big data” requires collaboration and the formation of large consortia funded by public–private partnerships that can overcome the diminishing resources of funding bodies and break the traditional barriers to multidisciplinary and inter-institutional research (13). Academic veterinarians and physicians need to communicate, conquer their silo guardians (14), and embrace new and more effective methods of working. Collaboration in “Comparative Medicine” will allow us to collect “big data,” which can be analyzed and “mined” using computational, bioinformatic (15), and “machine learning” techniques (16). The true value of large amounts of data hinges on the ability of researchers to put aside their paranoia and share data, methodologies, and findings in an open setting and allow free and unrestricted access to their databases (17). This strategy could change the bench to bedside paradigm and facilitate translational research. Fortunately, some progress is already being made in this area. Various consortia have been formed to take a One Health approach to disease prediction, control, and prevention (18). Of course, it is important to point out that this concept is not new. Rudolf Virchow the Father of Modern Pathology and Sir William Osler the Father of Modern Medicine were both outspoken advocates of the concept. However, these pioneers did not have access to the powerful genomic and post-genomic tools that we possess today. Genomic medicine has the potential to solve many of the unanswered questions in medicine and biology. However, the promise and success of genomic medicine will depend on rigorous comparative effectiveness research to compare outcomes for genome-based applications in practice to traditional non-genome-based approaches. This approach will be essential for assessing the evidence that will be used to enhance knowledge-driven practice of medicine in the twenty-first century (19).

## Exploiting the Power of Genomic Medicine

“Comparative Medicine” indeed is the medicine of the future and is paving the way forward for “personalized medicine” (20). However, “Comparative Medicine” in the twenty-first century should not solely focus on similarities between disease mechanisms in different species. In the era of genomic and post-genomic medicine, we have the opportunity to integrate epidemiological, clinical, and genetic information into sophisticated biomimetic virtual systems, which can help us develop sensitive *in silico* tools for modeling disease mechanisms by capitalizing on recent advances in genomic medicine. We need to take advantage of spontaneous analogs of human disease in companion animals to learn more about the pathogenesis of multifactorial diseases (11). Closer alignment of human and veterinary medicine and better communication between veterinary, medical, and biomedical sciences will help us achieve this challenge.

## A New Communication Channel

The veterinary and medical professions will face many new challenges in the twenty-first century. However, many of the challenges that will face these two communities are the same. Age-related diseases including cancer, neurodegenerative diseases, cardiovascular diseases, diseases of bones and joints (e.g., osteoarthritis, osteoporosis), and metabolic diseases (e.g., diabetes and insulin resistance) are common to humans and animals. Age-related diseases, emerging infectious diseases, and environmental threats are “One Health” challenges that need to be tackled using collaborative and interdisciplinary strategies. The convergence of human and animal health requires appropriate channels of communication. One of these channels of communication is Frontiers in Veterinary Science, a new journal developed by Frontiers Media S.A. and Nature Publishing Group (NPG). This open access journal is dedicated to the communication, discussion, and dissemination of all aspects of veterinary research. The journal’s core mission in animal health embraces the One Health concept in all of its specialty sections. “Comparative Medicine” is a specialty section of Frontiers in Veterinary Science devoted to the publication and dissemination of basic and translational research that focuses on laboratory animals and animal models, including experimental rodent and primate models, small and large domestic animal models of naturally occurring disease (inherited or acquired), and comparative anatomy, physiology, and immunology, including vaccines and immunization. This section has a broad scope that includes all animal species (including humans) and aims to encourage publication of comparative studies that address fundamental questions relating to One Health and One Medicine using molecular, cellular, tissue, and organ models. Our mission is to take a comparative approach to structure, function, and disease in animals and enhance our understanding of human health and disease. Multidisciplinary studies that tackle parallel diseases in humans and animals by engaging both veterinary and medical sciences are especially welcome. We also welcome papers that critically evaluate animal models and assess their suitability as models for human disease.

## Future Perspectives

This is an exciting time to be involved in “Comparative Medicine” research. We have the opportunity to take advantage of post-genomic technologies and advances in analytical techniques and develop better and more representative animal models for studying disease. The “One Health, One Medicine” concept is still evolving and it is hoped that in the twenty-first century it will realize its full potential. We therefore challenge and invite all the relevant stakeholders to contribute to this exciting section of “Comparative Medicine” section of Frontiers in Veterinary Science to disseminate new knowledge for the mutual benefit humans and animals.

## Conflict of Interest Statement

The author declares that the research was conducted in the absence of any commercial or financial relationships that could be construed as a potential conflict of interest.
